# Accuracy of Dose-Saving Artificial-Intelligence-Based 3D Angiography (3DA) for Grading of Intracranial Artery Stenoses: Preliminary Findings

**DOI:** 10.3390/diagnostics13040712

**Published:** 2023-02-14

**Authors:** Stefan Lang, Philip Hoelter, Manuel Alexander Schmidt, Anne Mrochen, Joji Kuramatsu, Christian Kaethner, Philipp Roser, Markus Kowarschik, Arnd Doerfler

**Affiliations:** 1 Department of Neuroradiology, University Hospital of Erlangen-Nuremberg, 91054 Erlangen, Germany; 2 Department of Neurology, University Hospital of Erlangen-Nuremberg, 91054 Erlangen, Germany; 3 Siemens Healthcare GmbH, Advanced Therapies, Innovation, Siemensstraße 1, 91301 Forchheim, Germany

**Keywords:** artificial intelligence, 3D angiography, deep learning, dose reduction, flat-detector computed tomography (FD-CT), intracranial artery stenosis, innovative postprocessing

## Abstract

Background and purpose: Based on artificial intelligence (AI), 3D angiography (3DA) is a novel postprocessing algorithm for “DSA-like” 3D imaging of cerebral vasculature. Because 3DA requires neither mask runs nor digital subtraction as the current standard 3D-DSA does, it has the potential to cut the patient dose by 50%. The object was to evaluate 3DA’s diagnostic value for visualization of intracranial artery stenoses (IAS) compared to 3D-DSA. Materials and methods: 3D-DSA datasets of IAS (n_IAS_ = 10) were postprocessed using conventional and prototype software (Siemens Healthineers AG, Erlangen, Germany). Matching reconstructions were assessed by two experienced neuroradiologists in consensus reading, considering image quality (IQ), vessel diameters (VD_1/2_), vessel-geometry index (VGI = VD_1_/VD_2_), and specific qualitative/quantitative parameters of IAS (e.g., location, visual IAS grading [low-/medium-/high-grade] and intra-/poststenotic diameters [d_intra-/poststenotic_ in mm]). Using the NASCET criteria, the percentual degree of luminal restriction was calculated. Results: In total, 20 angiographic 3D volumes (n_3DA_ = 10; n_3D-DSA_ = 10) were successfully reconstructed with equivalent IQ. Assessment of the vessel geometry in 3DA datasets did not differ significantly from 3D-DSA (VD_1_: *r* = 0.994, *p* = 0.0001; VD_2_:*r* = 0.994, *p* = 0.0001; VGI: *r* = 0.899, *p* = 0.0001). Qualitative analysis of IAS location (3DA/3D-DSA:n_ICA/C4_ = 1, n_ICA/C7_ = 1, n_MCA/M1_ = 4, n_VA/V4_ = 2, n_BA_ = 2) and the visual IAS grading (3DA/3D-DSA:n_low-grade_ = 3, n_medium-grade_ = 5, n_high-grade_ = 2) revealed identical results for 3DA and 3D-DSA, respectively. Quantitative IAS assessment showed a strong correlation regarding intra-/poststenotic diameters (r_dintrastenotic_ = 0.995, p_dintrastenotic_ = 0.0001; r_dpoststenotic_ = 0.995, p_dpoststenotic_ = 0.0001) and the percentual degree of luminal restriction (r_NASCET 3DA_ = 0.981; p_NASCET 3DA_ = 0.0001). Conclusions: The AI-based 3DA is a resilient algorithm for the visualization of IAS and shows comparable results to 3D-DSA. Hence, 3DA is a promising new method that allows a considerable patient-dose reduction, and its clinical implementation would be highly desirable.

## 1. Introduction

Atherosclerosis is considered the most important cause of the development of intracranial artery stenosis (IAS) [1,2] and accounts for approximately 8–37% of acute ischemic strokes worldwide [3,4,5,6,7,8,9]. Although non-invasive doppler sonography and CT and MR angiography represent valuable first-line diagnostics of IAS in the clinical routine [10,11,12,13,14,15,16], (because of its invasiveness) strictly indicated digital subtraction angiography (DSA) is the gold standard not only for providing secure evidence and grading of IAS but also for precise visualization of the vessel lumen [17,18].

Yet, IAS is by far not as complex as cerebral arteriovenous malformations (AVMs), and IAS grading by exclusive assessment of two-dimensional DSA (2D-DSA) series is potentially inadequate because intrastenotic measurements are frequently restricted to monoplanar images. Moreover, the evaluation of intracranial arteries is characterized by very small diameters and definitely suffers from a relevant measurement inaccuracy. As a consequence, 2D-DSA on its own is vulnerable to over- or underestimation of the stenotic dimensions and might be associated with incorrect therapeutic decisions in clinically symptomatic patients [19]. Thus, three-dimensional DSA volumes (3D-DSA) are obligatory for correct measurement and grading of IAS: 3D-DSA provides the opportunity to visualize the stenotic vessel segment at any angle and allows precise quantitative assessment. From a technical view, 3D-DSA relies—comparable with the principles of conventional 2D-DSA—on digital subtraction of a non-contrast-enhanced mask volume from a contrast-enhanced volume in order to generate angiographic 3D volumes without (e.g., osseous) overlap [20]. Because of its simple acquisition and postprocessing, 3D-DSA is currently seen as an essential part of DSA in the clinical routine and is considered highly reliable.

Nevertheless, the effective patient dose for a non-collimated 3D-DSA acquisition is 0.9 mSv and accounts for a relevant radiation dose of the entire examination [21]. Because 3D-DSA is one of the most frequent applications in the field of interventional neuroradiology, an optimization of its required radiation dose would be strongly desirable. In particular, the mask run, as a substantial component of the subtraction technique, accounts for approximately 50% of a 3D-DSA’s total radiation dose without actual information on vasculature. Hence, the mask run, especially, bears enormous potential for reduction in 3D-DSA’s radiation dose.

In this context, innovative postprocessing techniques using artificial intelligence (AI) for dose-reduced 3D angiographies (3DA) have been recently developed [22,23]. These (prototypical) algorithms allow a “DSA-like“ visualization of vessels but do not require mask runs. On the contrary, these algorithms exclusively operate with contrast-enhanced 3D volumes and are based on a deep neuronal network that is trained to classify different types of tissue (e.g., vasculature vs. bone vs. soft tissue [22] or vasculature vs. non-vasculature [20]). Until now, such algorithms have been primarily applied for the visualization of normal vasculature, cerebral aneurysms (CAs), AVMs, and fistulas (dAVFs) [22,23]. Both algorithms turned out to be diagnostic in these cases and were able to create high-resolution 3D angiographies for these pathologies.

However, IAS visualization can be extremely challenging due to low contrast intensities within the stenotic segment and associated vessel wall calcifications. Because there is no evidence of 3DA’s qualification in the field of IAS so far, we want to present our experience in visualization of IAS with a prototypical AI-based 3DA algorithm which classifies tissue also in a binary way (as previously described by Lang et al. [20,23]). Moreover, we want to evaluate the diagnostic value of 3DA reconstructions for IAS by comparison with the current standard technique, 3D-DSA.

## 2. Methods

### 2.1. Patient Selection

We screened our angiographic database (2018-2022) for consecutive patients presenting with intracranial artery stenosis and having received a 3D-DSA with diagnostic quality in order to evaluate the precise IAS grade and the indication for endovascular treatment, respectively. In a retrospective analysis, 3D-DSA datasets from 10 patients (age_mean_ ± SD = 60.1 ± 17.12 years; *n*_female_ = 4, *n*_male_ = 6) with untreated intracranial artery stenosis (*n*_IAS_ = 10) were selected and analyzed. The considered datasets for visualization of the anterior and posterior circulation (*n*_anterior circulation_ = 6; *n*_posterior circulation_ = 4) have been acquired in a standardized technique by contrast medium application via a diagnostic catheter positioned within the proximal internal carotid artery (ICA) or vertebral artery (VA), respectively. Written, informed consent was obtained from all patients enrolled. The study was performed according to the Declaration of Helsinki and the European Guidelines for Good Clinical Practice. Additional ethical review was not required for participation in this retrospective analysis in accordance with local legislation (BayKrG Section 27, paragraph 4) and institutional requirements.

### 2.2. Three-Dimensional Angiography 

The current standard to visualize vasculature in an unobstructed way is to perform a subtraction of a non-contrast-enhanced (“mask run”) from a contrast-enhanced volume (“fill run”), whereas the AI-based 3DA aims at the separation of vascular information to generate a 3D-DSA-like volume via an innovative postprocessing algorithm demanding a “fill run” only. 

The core of the AI-based method can be formulated as a binary classification problem: distinguishing vascular from non-vascular structures. To solve this problem, a machine learning-based approach was chosen incorporating the use of a deep convolutional neural network [24] and specifically trained for this task. The training of the network, which was carried out prior to this evaluation, entailed the presentation of contrast-enhanced volumes of cerebral vasculature (with and without digital subtraction) to the network. By this, it can be ensured that the network inherently learns to focus on the removal of the distinguishing information between the respective volumes, i.e., the non-vascular information, and to retain the vascular information. As a result, a 3D-segmentation mask featuring the determined vascular structures is generated. The segmentation mask can then be used on the data acquired during the fill run to calculate a 3D-DSA-like volume.

In terms of the network architecture, a feedforward 3D convolutional neural network has been chosen. The choice was driven by aiming for a reasonable trade-off between the runtime during application and the resulting quality of the classification process. In addition to this, it was important that volumes of arbitrary size, e.g., 512 × 512 × 512 voxels, could be used. The used network includes 4 convolutional layers with 8 filters each. The kernel size is 5 × 5 × 5 voxels. To include more contextual information during the classification process, while keeping the memory requirements low, dilated convolutions, as shown for example for a 1D scenario in [25], but also applicable to multidimensional approaches, were chosen to be incorporated. The dilation rate was set to 2. In the final layer of the network, the kernel size is 3 × 3 × 3 voxels with 1 result filter. In terms of the activation function, rectified linear unit (ReLu) activations were selected and normalized via batch normalization [26]. As a loss function, the binary cross entropy was set. Instead of a classical stochastic gradient descent optimization, the Adam optimization [27] approach with a batch size of 64 and a base learning rate of 0.01 was chosen.

The training of the prototypical AI-based 3DA algorithm was conducted on the basis of 98 conventionally acquired 3D-DSA datasets (free from metal as well as motion artifacts to ensure a high level of consistency) covering various conditions (e.g., datasets for three-dimensional visualization of cerebral aneurysms, AVMs, dAVFs, etc.) to prevent an algorithm’s overfitting during the training process. The presence of contrast-enhanced fill runs and the processed 3D-DSA volumes in the training data and their presentation to the network to be trained allowed for a suitable classification of the respective structures. The parameters used during the training were based on hyperparameter tuning. As an additional measure to increase the robustness of the algorithm, a patch-based training approach with randomly selected patches with a size of 31 × 31 × 31 voxels was chosen. This size represented the minimum input size for the neuronal network, and thus resulted in a single voxel response. Even though patches were used during the training, the application of the prototypical 3DA method is based on the processing of an entire volume. Testing and validation of 3DA were carried out multimodally with separate 3D-DSA datasets not involved in the training process (e.g., visualizing normal vasculature, cerebral aneurysms, AVMs, dAVFs, etc.). Essentially, neither datasets used for the prototype’s training nor those used for the prototype’s validation have been taken into account for the present evaluation of 3DA in the context of IAS. Finally, only the prototype’s successful validation allowed its application as part of this publication.

### 2.3. Data Acquisition and Postprocessing

Acquisition of 3D-DSA was performed on a biplane flat panel detector angiographic system (Artis zee biplane; Siemens Healthineers AG, Erlangen, Germany). A 5F catheter was positioned in the proximal internal carotid artery (ICA) or vertebral artery (VA) to obtain 3D-DSA datasets by using a standard protocol for acquisition as provided by the manufacturer (5s 3D-DSA): An initial rotational scan (native mask run) was followed by a second rotational scan (contrast-enhanced fill run) of 5s each. Each run yields 133 projections (rotational angle: 200°). The detector dose per projection image is selected as 0.36 μGy (70 kV, 1240 × 960 detector elements with 2-by-2 binning of pixels, projection on a 30 × 40 cm flat panel size, increment of 1.5°/frame, frame-rate of 30 frames/s). According to the protocol, a manual injection of contrast was initiated 1s before the beginning of the fill run, maintained for 6 s, and stopped after the C-arm system covered the complete rotation angle. The total contrast volume was 15 mL (Iopamidol, Imeron 300; Bracco, Milan/Italy).

Both mask- and fill runs of the acquired 3D-DSA datasets were transferred to a dedicated workstation (*syngo* X-Workplace; Siemens Healthineers AG, Erlangen/Germany) running both commercially available software for conventional 3D-DSA postprocessing and an additional software prototype plug-in for the 3DA postprocessing. Reconstruction of the 3D-DSA volumes was performed using data derived from both runs, whereas reconstruction of 3DA volumes was accomplished using only data from the fill runs. 

According to standardization, we used conventional reconstruction parameters for both 3DA and 3D-DSA (kernel type: “edge-enhanced”; characteristics: “smooth”; 512 × 512 image matrix).

### 2.4. Image Evaluation

The image quality of all datasets was evaluated for parameters that could compromise the diagnostic value using a 5-fold grading scale (see Table 1). The 3D-DSA and 3DA reconstructions were assessed in consensus reading by 2 experienced neuroradiologists (with 8 and 12 years of clinical experience) blinded to the type of reconstruction (based on either the subtraction technique or the AI algorithm).

### 2.5. Assessment of 3DA and 3D-DSA Reconstructions

#### 2.5.1. Vessel-Geometry Index (VGI)

**Figure 1:**
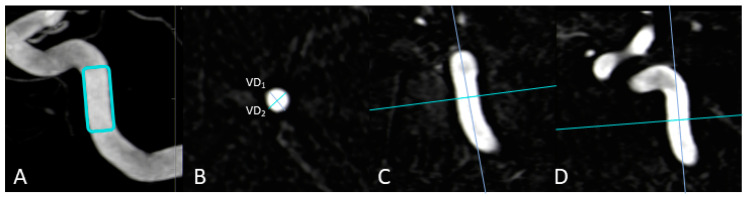
Exemplary measurement of a vessel-geometry-index (VGI) in a 3D-DSA dataset. In this anterior circulation case, the vessel diameters (VD_1_; VD_2_) of the ICA (**B**) are determined using multiplanar reconstructions at the level of the C4 segment (**A**) in an axial plane (**B**) after precise alignment of the vessel (**C**,**D**). In the end, the ratio of VD1 to VD_2_ enables the estimation of the VGI (VGI = VD_1_/VD_2_).

As previously described in [20], the maximum transversal diameters of the injection vessels (in mm; VD_1_/VD_2_) have been measured in multiplanar reconstructions in all 3DA- and 3D-DSA reconstructions. Taking the three-dimensionality of these datasets into account, the ratio of VD_1_ and VD_2_ was defined as the “vessel-geometry index” (“VGI” = VD_1_/VD_2_). Measurements for vessels of the anterior and posterior circulation were performed at the C4 segment of the ICA and at the V4 segment of the VA, respectively. See also Figure 1 for further illustration.

#### 2.5.2. Intracranial Artery Stenosis

The degree of agreement between the 3DA and 3D-DSA datasets of the IAS was evaluated with a compound of MPR-/MIP-/VRT-datasets from both types of reconstructions.

As qualitative parameters, firstly the location of IAS (e.g., M1 for the proximal segment of the middle cerebral artery [MCA], etc.) and secondly a visual grading of the IAS (e.g., low-grade IAS in cases of only minimal narrowing of the arterial lumen, medium-grade IAS in cases of moderate narrowing of the arterial lumen and high-grade IAS in cases of severe narrowing of the arterial lumen) were determined for all datasets. Analogous to the North American Symptomatic Carotid Endarterectomy Trial (NASCET) [28], the narrowest intrastenotic diameter (d_intrastenotic_ in mm) and the normal vessel diameter distal to the stenosis (d_poststenotic_ in mm) were assessed as quantitative parameters for calculation of the percentual degree of luminal restriction [(d_poststenotic_ − d_intrastenotic_)/d_poststenotic_ × 100].

### 2.6. Statistical Analysis

Statistical analysis was performed using commercially available software (SPSS Statistics Version 20, IBM, Chicago, IL, USA).

Because of their non-continuous character, qualitative parameters (e.g., location of IAS and visual grading of the IAS) were analyzed by use of descriptive statistics. 

Quantitative parameters from both groups (e.g., vessel diameters, vessel-geometry indices, intrastenotic diameter [d_intrastenotic_], vessel diameter distal to the stenosis [d_poststenotic_], and percentual degree of luminal restriction were tested for normal distribution by using the D’Agostino–Pearson test (if *p* > 0.05 normality was accepted) and were compared by using the Pearson correlation coefficient (r) and a paired *t*-test (p), respectively.

## 3. Results

### 3.1. Image Quality

All 3DA and 3D-DSA reconstructions (*n*_total_ = 20) were of diagnostic quality. There was no case with a relevant reduction in the IQ (3DA: *n*_excellent_ = 10; 3D-DSA: *n*_excellent_ = 10).

### 3.2. Qualitative and Quantitative Assessment of 3D-DSA and 3DA Reconstructions

Vessel-Geometry-Index (VGI)

Measurement of vessel diameters was successfully performed for all 3DA and 3D-DSA reconstructions, (*n*_3DA_ = 20, *n*_3D-DSA_ = 20). Neither the acquired values for VD_1_/VD_2_ nor the calculated values for the corresponding VGI showed significant differences (r_VD1_ = 0.994; p_VD1_ = 0.0001; r_VD2_ = 0.994; p_VD2_ = 0.0001; r_VGI_ = 0.899; p_VGI_ = 0.0001). 

See Table 2 for further details.

### 3.3. Intracranial Artery Stenosis

Qualitative assessment of the corresponding 3DA and 3D DSA datasets revealed equivalent results concerning the location of IAS (3DA: n_ICA/C4_ = 1, n_ICA/C7_ = 1, n_MCA/M1_ = 4, n_VA/V4_ = 2, n_BA_ = 2; 3D DSA: n_ICA/C4_ = 1, n_ICA/C7_ = 1, n_MCA/M1_ = 4, n_VA/V4_ = 2, n_BA_ = 2) and visual IAS-grading (3DA: n_low-grade_ = 3, n_medium-grade_ = 5, n_high-grade_ = 2; 3D DSA: n_low-grade_ = 3, n_medium-grade_ = 5, n_high-grade_ = 2).

Quantitative assessment of the corresponding 3DA and 3D DSA datasets showed a strong correlation with regards to the intra- and poststenotic diameters (r_dintrastenotic_ = 0.995, p_dintrastenotic_ = 0.0001; r_dpoststenotic_ = 0.995, p_dpoststenotic_ = 0.0001). Consecutively, also calculation of the percentual degree of luminal restriction according to the NASCET criteria correlated well for both reconstruction types (r_NASCET_ = 0.981; p_NASCET_ = 0.0001).

**Figure 2:**
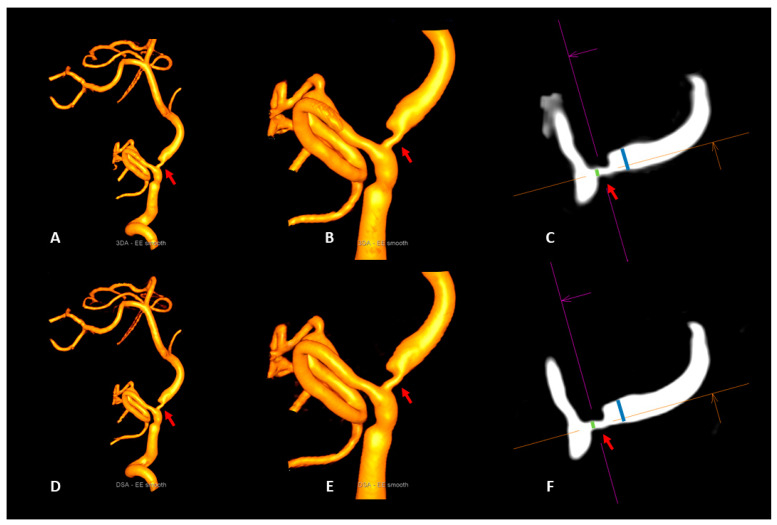
Illustrative case 1. Visualization of a vertebrobasilar stenosis (red arrows) with the artificial intelligence-based 3DA (**A**–**C**) and 3D-DSA (**D**–**F**). 3DA (**A**,**B**) provides a 3D-DSA (**D**,**E**) comparable visibility of the stenotic segment (red arrows) in the volume-rendered reconstructions. Due to the high level of correspondence of the measurements of the intra- and poststenotic diameters in multiplanar reconstructions of 3DA (**C**) and 3D-DSA (**F**), the calculation of the percentual degree of luminal restriction revealed a 64% stenosis of the vertebrobasilar junction for both reconstruction types (NASCET_3DA_ = 63.9%; NASCET_3D-DSA_ = 63.6%).

**Figure 3:**
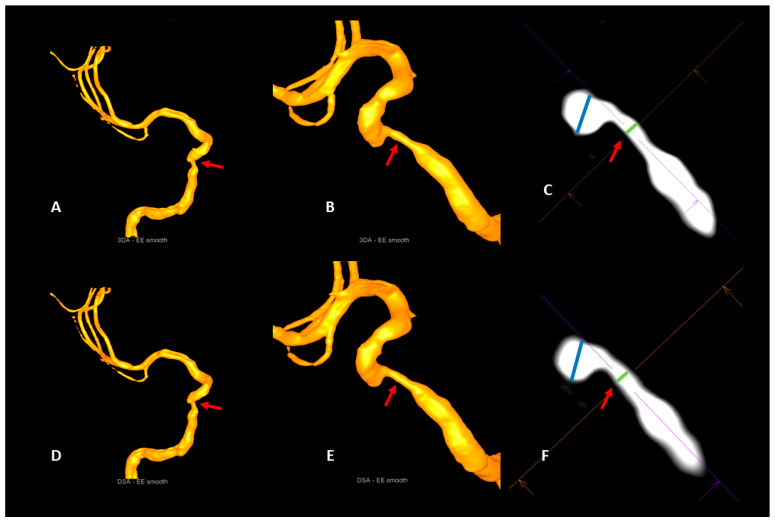
Illustrative case 2. Visualization of a stenosis of the cavernous segment of the right internal carotid artery (red arrows) with the artificial intelligence-based 3DA (**A**–**C**) and 3D-DSA (**D**–**F**). Comparable with 3D-DSA (**D**,**E**), 3DA (**A**,**B**) provides a clear visualization of the stenosis (red arrows) in the volume-rendered reconstructions. The measurements of the intra- and poststenotic diameters in multiplanar reconstructions of 3DA (**C**) and 3D-DSA (**F**) do not show a relevant deviation from each other. As a consequence, the calculation of the percentual degrees of luminal restriction in the 3DA (NASCET_3DA_ = 70.5%) and the 3D-DSA (NASCET_3D-DSA_ = 69.5%) reconstruction shows a strong accordance.

Please see Table 2 for further details and Figure 2 and Figure 3 for illustrative cases.

## 4. Discussion

Three-dimensional imaging of neurovascular pathologies as part of cerebral catheter angiography is substantial for precise diagnostics and adequate therapy. Even though 3D-DSA is currently considered the gold standard for this purpose, application of the subtraction technique is linked with relevant disadvantages: 3D-DSA always requires the acquisition of both a native and a contrast-enhanced volume for the separation of vasculature. On the one hand, this procedure leads to a doubled radiation exposure for patients. On the other hand, the actual image quality strongly depends on the efficiency of the data registration and is highly susceptible to motion artifacts. Therefore, AI-based algorithms relying on a single contrast-enhanced volume for “DSA-like” 3D imaging of vasculature are a promising new development. In particular, the application of the AI-based 3DA cuts down not only radiation doses but also improves image quality in cases of patient motion [22]. By now, the qualification of such AI-based algorithms has already been demonstrated for a variety of neurovascular pathologies (e.g., AVMs, CAs, etc.) [20,22,23]. However, data on 3DA’s diagnostic value for visualization of IAS is still pending. Consequently, an evaluation of 3DA focusing on IAS should be pursued, not least because of its clinical importance. 

In our series, all IAS datasets have been successfully reconstructed with the prototypical AI-based 3DA. Moreover, the quantitative and qualitative comparison of 3DA with the conventional 3D-DSA demonstrated equivalent visualization of IAS. Thus, the results of our analysis confirm existing data on AI-based algorithms for dose-reduced 3D imaging of vasculature. 

In 2018, Montoya et al. [22] were the first to describe an AI-based algorithm (“3D deep-learning angiography”/3D-DLA) that effectively extracts vascular structures by differentiating three types of tissue (vasculature/bone/soft tissue) for each image voxel. 3D-DLA had been trained and validated with 43 datasets. After that, 62 other datasets with cerebrovascular abnormalities were the object of the actual evaluation. For this purpose, both subtraction technique-based and AI-based reconstructions have been assessed, for example, with respect to vasculature classification accuracy and quality of bone removal. 3D-DLA turned out to be highly reliable and precise. As another key finding, the application of 3D-DLA for blurred datasets improved overall image quality and in particular the visibility of small vessels. Moreover—as a consequence of the “single-run design” of 3D-DLA—Montoya et al. stated that their algorithm helps to significantly reduce the radiation dose. Even though an explicit evaluation of IAS has not been performed in [22], our data support Montoya’s statements concerning the high potential of AI-based approaches to optimize patient dose and image quality.

In 2019, Lang et al. [20] reported on a similar AI-based algorithm (3D angiography/“3DA”) that also enables “DSA-like” visualization of vasculature by classifying two different types of tissue (vasculature/non-vasculature) for each image voxel. This algorithm was trained and validated with 98 datasets. Initially, 15 datasets without pathological findings [20] and later 30 datasets with neurovascular pathologies (AVMs, dAVFs, CAs) [23] were quantitatively and qualitatively evaluated. In both series, subtraction technique and AI-based reconstructions were compared with regard to the quality of vessel visualization. Similar to Montoya’s results, a high level of reliability and accuracy was demonstrated for 3DA in cases of normal and pathologic vasculature. Consequently, the manuscripts’ central statement is that 3DA’s “single-run design” offers a considerable reduction in the patient dose and should find a direct path to clinical implementation. Although focusing on IAS, our data indicate 3DA’s efficiency in terms of dose reduction and improving image quality as well. Therefore, we believe that—not least because our proposed 3DA algorithm is also characterized by a binary design comparable with [20,23]—our results fit in well with existing data.

Beyond AI-based algorithms, thresholding of contrast-enhanced datasets can also provide a “DSA-like” 3D rotational angiography (3D-RA) and supersede the acquisition of a native mask run for application of the subtraction technique [29]. Because of the “single-run design” comparable with 3D-DLA or 3DA, approximately 50% of the usual 3D-DSA dose can be saved in case of 3D-RA’s application, and the negative effects of the DSA-typical problem of intersweep motion can be completely eliminated. On the basis of reliability and accuracy in cases of cerebral aneurysms, 3D-RA has already proven its diagnostic value. From a technical point of view, 3D-RA works by modifying thresholds to separate vascular from nonvascular structures [30,31]. Thus, imaging of low-opacified vessels is prone to error [32]. In detail, visualization of very small vessels cannot securely be provided by 3D-RA. Hence, 3D-RA seems to be of limited applicability in cases of IAS, because small vascular proportions are characteristic of IAS and associated with low contrast, especially in high-grade stenoses. In contrast, AI-based algorithms appear unimpressive as 3DA has demonstrated an accurate visualization of small perforating arteries as well [20]. Therefore, our results indicate that the use of 3D-RA should focus on vascular structures with an expectable high contrast (e.g., aneurysms), whereas 3DA is unconditionally applicable for all neurovascular pathologies. 

Apart from AI-based and thresholding algorithms, variations of scanning parameters as the most obvious approach to achieve effective dose reductions have been described as useful for subtraction-based 3D imaging [33,34]. In this context, especially the “dose per frame” and “scanning time” attracted attention because downscaling of dose per frame up to 70% (from 0.36 µGy/f towards 0.10 µGy/f) still generated useful image quality in an animal model. Finally, Pearl et al. identified a smart combination of parameters (5s and 0.24 µGy/frame) providing tolerable image quality for patients with intracranial aneurysms and an estimated dose reduction of 30%. Even though Pearl et al. rated these deviations as acceptable, any manipulation of the standard protocol was associated with relevant deviations regarding the vascular geometry. Due to the natural susceptibility of (high-grade) IAS for over- or underestimation, Pearl’s low-dose protocols do not seem appropriate for IAS. However, we fully agree with the hypothesis [20,23] that Pearl’s low-dose approach is highly relevant for further development of AI-based algorithms: Simultaneous application of low-dose protocols and AI-based algorithms (regardless of a binary or ternary design) has potentially a far greater impact on dose reduction for 3D imaging of vasculature than any of these methods on their own. Hence, future research should prioritize the development of clinically applicable dose-reduced single-run protocols.

## 5. Limitations

Even though useable 3DA reconstructions of IAS have been realized for all considered datasets, our analysis has limitations. First, it was limited by the small sample size of IAS, the strict selection criteria for the considered datasets (e.g., selective catheter position for contrast application, no metal artifacts, etc.), and its evaluation design without an independent assessment of the reconstructions. Moreover, our analysis does not include volumes with compromised image quality (e.g., heavy motion artifacts, etc.) and low contrast (e.g., datasets with varying contrast media dilutions because of unexpected loss of the catheter position). Apart from this, our analysis exclusively evaluated one specific AI-based algorithm based on a specific neuronal network trained for binary classification of cerebral structures (vasculature vs. non-vasculature). Thus, a valid transfer of our results concerning the diagnostic value of our proposed 3DA algorithm can only be directly transferred to other algorithms with comparable technical characteristics (incl. training and validation conditions) [20,23]. As a consequence of these issues, further investigations should focus on a direct comparison between the existing AI-based algorithms to precisely evaluate the drawbacks of each algorithm and provide an independent evaluation design by preference. Moreover, these AI-based algorithms should prospectively cover both dual-volume datasets (e.g., coiled/clipped aneurysms) to allow dose savings also for postinterventional cases and volumes with compromised image quality to further extend 3DA’s field of application. Besides technical aspects and because of the high clinical relevance of lacunar ischemic strokes [35], future publications should also evaluate the diagnostic accuracy of such AI-based algorithms in the assessment of stenotic changes of small arteries (e.g., lenticulostriate perforators). 

## 6. Conclusions

Reconstructions of the proposed AI-based 3DA algorithm provide a reliable and accurate visualization of intracranial artery stenoses. In direct comparison with conventionally reconstructed 3D-DSA volumes, 3DA turned out to be equivalent for IAS imaging. Furthermore, 3DA cuts the effective patient dose by approximately 50% because of its single-run design. Thus, a broad clinical application of this innovative post-processing algorithm would be highly desirable. The concepts and results presented in this paper are based on research and are not commercially available.

## Figures and Tables

**Table 1:** Image Quality (IQ).

Grade_IQ_	Characteristics
4	excellent (high contrast, clear delineation of the stenosis, no artifacts)
3	good (high contrast; good delineation of the stenosis, minimal artifacts, e.g., due to movement)
2	compromised (e.g., noticeable movement artifacts and/or reduced homogeneity of the vessel contrast, delineation of the stenosis still acceptable)
1	heavily compromised (low contrast and/or strong movement artifacts, difficult delineation of the stenosis)
0	not diagnostic (no delineation of the stenosis)

**Table 2:** Vessel diameters (VD), vessel-geometry index (VGI), NASCET graduation of IAS.

Parameter	3DA	3D-DSA	*r*	*p*
VD1_IAS_	4.41 ± 0.69 mm	4.36 ± 0.64 mm	0.994	0.0001
VD2_IAS_	4.65 ± 0.63 mm	4.58 ± 0.61 mm	0.994	0.0001
VGI_IAS_	0.95 ± 0.03 mm	0.95 ± 0.03 mm	0.899	0.0001
d_poststenotic_	1.74 ± 0.58 mm	1.68 ± 0.53 mm	0.995	0.0001
d_intrastenotic_	3.12 ± 0.71 mm	3.04 ± 0.67 mm	0.993	0.0001
NASCET_IAS_	43 ± 0.16%	44 ± 0.15%	0.981	0.0001

3DA = 3D angiography; 3D-DSA = 3D digital-subtraction angiography; VD_1/2_ = vessel diameter_1/2_; IAS = intracranial artery stenosis; d_intra-/poststenotic_ = diameter_intra-/poststenotic_; NASCET = North American Symptomatic Carotid Endarterectomy Trial.

## Data Availability

The data presented in this study are available on request from the corresponding author.
